# Arboviruses Beyond Traditional Boundaries: Climate-Driven Vector Expansion and Emerging One Health Risks

**DOI:** 10.3390/pathogens15070683

**Published:** 2026-06-27

**Authors:** Miray Tonk-Rügen, Ludwig E. Hoelzle, Alejandro Cabezas-Cruz

**Affiliations:** 1 Institute for Insect Biotechnology, Justus Liebig University of Giessen, 35392 Giessen, Germany; 2 Department of Livestock Infectiology and Environmental Hygiene, Institute of Animal Science, University of Hohenheim, 70593 Stuttgart, Germany; ludwig.hoelzle@uni-hohenheim.de; 3 HoLMiR—Hohenheim Center for Livestock Microbiome Research, University of Hohenheim, 70593 Stuttgart, Germany; 4 ANSES, INRAE, Ecole Nationale Vétérinaire d’Alfort, UMR BIPAR, Laboratoire de Santé Animale, F-94700 Maisons-Alfort, France; alejandro.cabezas@vet-alfort.fr

The recent publication by Larska et al. describing the first detection of bluetongue virus serotype 3 (BTV-3) in Poland in a European bison (*Bison bonasus*) represents more than an isolated wildlife case; it highlights how wildlife surveillance can provide early warning of the ecological changes driving climate-sensitive arbovirus emergence across Europe [1]. The case, detected in Wolin National Park close to the German–Polish border, involved a fatal hemorrhagic disease in a European bison, with BTV-3 confirmed in the animal and in a pool of blood-fed *Culicoides punctatus* collected near the enclosure two weeks later [1]. Phylogenetic analysis linked the Polish isolate closely to BTV-3 strains circulating in the Netherlands, Germany, and Portugal, supporting the view that this virus is part of a wider European epizootic wave rather than a geographically restricted event [1]. Importantly, the study highlights the value of wildlife as sentinels of emerging infections that may remain undetected when surveillance focuses primarily on domestic livestock.

Bluetongue virus is not a zoonotic pathogen, but it is highly relevant to One Health because it connects vector ecology, livestock production, wildlife conservation, animal welfare, international trade, and climate-sensitive disease emergence. The emergence and rapid spread of BTV-3 in northern and central Europe since its first detection in the Netherlands in September 2023 illustrate how quickly arboviruses can establish and expand when ecological conditions become favorable [2,3,4]. Culicoides biting midges, the biological vectors of BTV, are strongly influenced by meteorological and ecological factors, including temperature, relative humidity, rainfall, wind-assisted dispersal, and the availability and proximity of vertebrate hosts [5,6,7,8]. Within suitable thermal limits, warmer conditions can prolong seasonal vector activity, shorten viral replication time within vectors, and increase the probability of transmission during periods that were previously less suitable [8,9,10,11]. In the study, Culicoides were actively feeding until the end of October, a detail that is particularly relevant in the context of longer autumn transmission windows [1]. Together, these observations illustrate how environmental change can reshape vector ecology and create opportunities for pathogen emergence. Yet the establishment of transmission cycles ultimately depends not only on vector presence but also on the ecological compatibility among pathogens, vectors, and vertebrate hosts (Figure 1).

The implications of the Polish BTV-3 case extend well beyond bluetongue. Across Europe, climate change is altering the distribution, abundance, and seasonal activity of several arthropod vectors, including mosquitoes, ticks, and biting midges [12]. The World Health Organization emphasizes that vector-borne diseases account for more than 17% of all infectious diseases globally and cause more than 700,000 deaths annually; it also notes that climate change, global travel, trade, urbanization, and vector adaptation contribute to the spread of these diseases [13]. In Europe, this shift is now visible through the increasing frequency and geographic expansion of locally acquired mosquito-borne arbovirus infections, including West Nile virus infection, dengue, and chikungunya [14,15,16,17]. ECDC reported that Europe is experiencing longer and more intense transmission seasons for mosquito-borne diseases, driven by rising temperatures, longer summers, milder winters, and altered rainfall patterns [18]. *Aedes albopictus*, a competent vector for dengue, chikungunya, and Zika viruses, is now established in 16 European countries and 369 regions, compared with 114 regions about a decade earlier [18,19,20]. In 2024, Europe recorded 304 locally acquired dengue cases and 1436 West Nile virus infections across 212 regions in 19 countries, emphasizing that arbovirus emergence is no longer restricted to traditionally endemic regions [20].

Ticks are undergoing similar ecological shifts. Climate warming, land-use change, host population dynamics, and increased human–wildlife contact are modifying the distribution, abundance, and seasonal activity of major European ticks, including *Ixodes ricinus*, *Dermacentor* spp., and *Hyalomma* spp. [21,22,23,24,25]. These ecological shifts may alter the risk of exposure to tick-borne pathogens, including arboviruses such as tick-borne encephalitis virus and Crimean-Congo hemorrhagic fever virus, as well as non-viral pathogens such as *Borrelia burgdorferi* sensu lato, *Theileria* spp., *Anaplasma phagocytophilum*, and *Babesia* spp. [21,22,26,27,28]. Recent reviews and modeling studies highlight that tick populations are expanding northwards and to higher altitudes in parts of Europe, while climate-favored species such as *Hyalomma marginatum* raise concern for the possible introduction of pathogens previously associated with warmer regions [21,23,24,27,29]. Therefore, surveillance and risk assessment for arboviruses and other vector-borne pathogens should not be limited to mosquitoes. A broader arthropod–vector perspective is required, integrating ticks and insects into surveillance and risk assessment frameworks.

The Polish BTV-3 bison case also emphasizes the importance of wildlife in arbovirus ecology. Wildlife species can function as sentinels, reservoirs, amplifiers, or dead-end hosts, depending on the pathogen and ecological context. However, wildlife remains one of the least integrated components of many surveillance systems, despite its potential to provide early signals of pathogen emergence and spread. European bison are of high conservation value, and disease outbreaks in such species can have implications beyond animal health, affecting biodiversity protection and rewilding initiatives. The detection of BTV-3 in a protected wildlife population, therefore, raises important questions: could wildlife-based surveillance provide earlier warning of emerging arboviruses than traditional livestock-focused monitoring systems? Can entomological surveillance detect viral circulation before clinical disease appears? Are veterinary and conservation authorities prepared for climate-sensitive arboviruses in free-ranging and semi-captive wildlife populations?

A One Health approach is essential because arbovirus emergence is shaped by the combined effects of climate, ecosystems, vectors, animals, and humans. The same environmental drivers that support BTV transmission by Culicoides can also favor mosquito-borne and tick-borne pathogens. Yet surveillance systems often remain fragmented: livestock diseases are monitored separately from human arboviruses, wildlife health data are limited, and vector surveillance is unevenly implemented across regions. Integrated surveillance should combine clinical reporting, serology, molecular diagnostics, vector trapping, pathogen screening, meteorological data, land-use information, and host movement data. Genomic epidemiology, as demonstrated by the phylogenetic analysis of BTV-3 in the Polish case, can further clarify routes of virus introduction and spread [1]. The main lesson from the BTV-3 emergence is not that climate change alone causes outbreaks. Rather, climate change increases ecological permissiveness, while animal movement, trade, windborne vector dispersal, land-use change, wildlife management, and surveillance determine where and when transmission becomes established and detected.

This complexity requires proactive risk management rather than reactive responses. Early warning systems should identify regions where vector activity, host susceptibility, and pathogen introduction risk overlap. Vaccination strategies for livestock, where available, should be combined with vector monitoring and transparent communication with farmers, veterinarians, wildlife managers, and public health authorities. For zoonotic arboviruses such as West Nile, dengue, chikungunya, tick-borne encephalitis, and Crimean–Congo hemorrhagic fever, public health preparedness must also include clinician awareness, laboratory capacity, international collaborations, and public education. Because arthropod vectors, animal movements, and arboviruses are not constrained by national borders, preparedness must also rely on transboundary collaboration, harmonized surveillance systems, and timely data sharing between neighboring countries.

In conclusion, the first detection of BTV-3 in a European bison in Poland should be viewed as a sentinel event in a broader European pattern of vector-borne disease emergence. Arboviruses are no longer distant threats confined to tropical or subtropical regions; they are increasingly part of Europe’s animal and human health reality. The expansion of ticks, mosquitoes, biting midges, and other arthropod vectors demands integrated surveillance, climate-informed risk modeling, and stronger collaboration among veterinary medicine, public health, entomology, parasitology, wildlife conservation, and environmental sciences. As the distributions of vectors, hosts, and pathogens continue to shift, wildlife, livestock, and human health surveillance can no longer operate in isolation. The Polish BTV-3 case demonstrates that understanding the epidemiology of emerging arboviruses will require not only monitoring environmental change but also recognizing wildlife as a critical component of early-warning systems for infectious disease emergence.

## Figures and Tables

**Figure 1:**
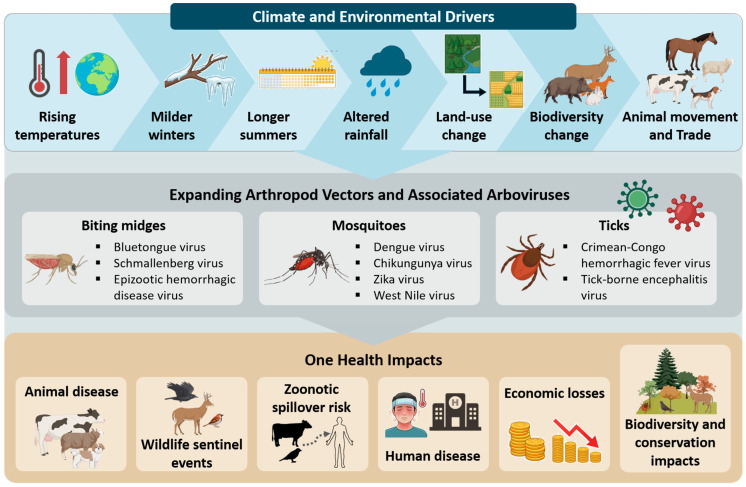
Climate-driven expansion of arthropod vectors and arbovirus transmission within a One Health framework. Rising temperatures, altered rainfall, land-use change, and animal movement can reshape the distribution and seasonal activity of biting midges, mosquitoes, and ticks. As these vectors expand, associated arboviruses increasingly threaten animal health, wildlife conservation, human health, economic stability, and biodiversity protection.
